# Human Cytomegalovirus-Induced Interleukin-10 Production Promotes the Proliferation of *Mycobacterium massiliense* in Macrophages

**DOI:** 10.3389/fimmu.2020.518605

**Published:** 2020-09-10

**Authors:** Hailian Quan, Jiyeon Kim, Yi Rang Na, Jung Heon Kim, Byoung-Jun Kim, Bum-Joon Kim, Jung Joo Hong, Eung Soo Hwang, Seung Hyeok Seok

**Affiliations:** ^1^Department of Microbiology and Immunology, Seoul National University College of Medicine, Seoul, South Korea; ^2^Institute of Endemic Disease, Seoul National University Medical Research Center, Seoul, South Korea; ^3^Global Center for Infectious Diseases, Seoul National University College of Medicine, Seoul, South Korea; ^4^Transdisciplinary Department of Medicine and Advanced Technology, Seoul National University Hospital, Seoul, South Korea; ^5^National Primate Research Center, Korea Research Institute of Bioscience and Biotechnology, Cheongju, South Korea; ^6^Department of Biomedical Sciences, Seoul National University College of Medicine, Seoul, South Korea

**Keywords:** human cytomegalovirus, macrophage, *Mycobacterium massiliense*, interleukin-10, non-tuberculous mycobacteria

## Abstract

Human cytomegalovirus (HCMV) exploits the interleukin-10 (IL-10) pathway as a part of its infection cycle through the manipulation of the host IL-10 signaling cascade. Based on its immunomodulatory nature, HCMV attenuates the host immune response and facilitates the progression of co-infection with other pathogens in an immune-competent host. To investigate the impact of HCMV infection on the burden of non-tuberculous mycobacteria (NTM), whose prevalence is growing rapidly worldwide, macrophages were infected with HCMV and further challenged with *Mycobacterium massiliense in vitro*. The results showed that HCMV infection significantly increased host IL-10 synthesis and promoted the proliferation of *M. massiliense* in an IL-10-dependent manner. Transcriptomic analysis revealed that HCMV infection dampened the regulatory pathways of interferon gamma (IFN-γ), tumor necrosis factor alpha (TNF-α), and interleukin-1 (IL-1), consequently abrogating the immune responses to *M. massiliense* coinfection in macrophages. These findings provide a mechanistic basis of how HCMV infection may facilitate the development of pathogenic NTM co-infection by upregulating IL-10 expression.

## Introduction

Human cytomegalovirus (HCMV) is a species-specific beta-herpesvirus that infects the majority of the world’s population (1). The general absence of HCMV disease imposes an extraordinarily large immunological burden on its infected host (2). The ability to establish and maintain a persistent infection in the presence of antiviral immunity requires the contribution of the capacity of HCMV to encode immune evasive proteins that alter cellular signaling and activation (3). The capacity of HCMV to successfully infect the host and cause disease is partially obtained from the cytomegalovirus-encoded human interleukin-10 (cmvIL-10) (4). The expression of cmvIL-10, encoded by the *UL111A* gene, during productive HCMV infection is known to upregulate host IL-10 production in monocytes via phosphatidylinositol 3-kinase (PI3K)/signal transducer and activator of transcription 3 (STAT3) signaling pathway. Meanwhile, macrophages are important sites for HCMV replication (5), and act as notable producers of IL-10 in our body (6). Being the most potent anti-inflammatory cytokine (7, 8), this led us to consider that HCMV infection in macrophages might facilitate co-infection of *Mycobacterium abscessus*, which proliferates mostly inside the macrophages and is the most drug-resistant species among the non-tuberculous mycobacteria (NTM) (9). Recent reports support this idea in that they have noticed the convergent epidemiology of tuberculosis and HCMV infection (10, 11). Therefore, we assessed the effect of HCMV infection on the proliferation of *Mycobacterium massiliense*, a *M. abscessus* subspecies, in macrophages in order to understand the participation of HCMV-mediated immune modulation as a risk factor for coinfection with NTM and a high burden of NTM.

## Materials and Methods

### Virus and Bacteria Strains

Human cytomegalovirus Towne (ATCC VR-977) and UL32-EGFP-HCMV-TB40E (ATCC VR-1578) strains were maintained as described previously (12, 13). *M. massiliense* CIP strain (ATCC 108297) was obtained from CIP (Collection of Institute Pasteur). *M. massiliense* was grown in Middlebrook 7H9 broth (BD Biosciences) supplemented with 0.2% glycerol (Sigma-Aldrich), 10% oleic acid-albumin-dextrose-catalase (OADC; Thermo Fisher Scientific), and 0.05% Tween 80 (Sigma-Aldrich). Cultures were incubated at 37°C with constant shaking (150 rpm) overnight to reach an optical density of 0.5–0.7 at 600 nm (OD600). Collected mycobacteria were homogenized and stored at −80°C.

### Cell Culture

The human acute monocytic leukemia THP-1 (ATCC TIB-202) cell line was obtained from the ATCC and maintained in RPMI media containing 10% FBS (Gibco) and 1% penicillin-streptomycin (PS; Gibco) at 37°C in a humidified atmosphere with 5% CO_2_. Differentiation of THP-1 cells into macrophages was performed by incubating the cells with 50 ng/mL phorbol 12-myristate 13-acetate (PMA) (Sigma-Aldrich) for 2 days, and then, the media was changed to that without 1% PS for 1 day. PMA-differentiated THP-1 cells (THP-1 macrophages) were infected with HCMV Towne (otherwise indicated) or TB40E strains (in case of analyzing GFP expression) at a multiplicity of infection of 10 (MOI = 10) for 3 h, and the media was changed to establish HCMV infection. In case of obtaining HCMV culture supernatant, THP-1 macrophages were infected with HCMV Towne for 3 h, and the media was changed and further incubated for 24 h before collecting culture supernatant. *M. massiliense* was infected at (MOI = 2) 24 h post-HCMV infection. After 1 h of *M. massiliense* infection, cells were washed with PBS 2 times to remove extracellular bacteria and cultured with complete media without antibiotics for 1∼3 days. For quantification of colony-forming units (CFUs), the infected cells were lysed in PBS containing 0.1% Triton X-100 (Sigma-Aldrich) and plated on LB agar.

Human peripheral blood mononuclear cells (PBMCs) were isolated from healthy donors (Approved IRB No. C-1306-0210494) by Ficoll-Hypaque Plus (GE Healthcare) gradient centrifugation. Freshly isolated PBMCs were seeded and infected with HCMV at a MOI of 10 for 24 h, and the media was changed to establish HCMV infection. *M. massiliense* was infected at (MOI = 2) 24 h post-HCMV infection.

### Recombinant Proteins and Neutralizing Antibodies

Recombinant cmvIL-10 was obtained from R&D systems. Purified viral HCMV IL-10 antibody (αcIL-10, polyclonal goat IgG, R&D Systems) and human IL-10 antibody (αhIL-10, clone 23738, R&D Systems) were used at 10 μg/mL to neutralize cIL-10 and hIL-10 protein. Human IL-10 receptor alpha neutralizing monoclonal antibody (αhIL-10R, clone 37607, R&D Systems) was used at 10 μg/mL. Corresponding isotype controls for neutralization experiments were obtained from R&D Systems.

### Immunofluorescence Staining

Cells were fixed in 4% paraformaldehyde (PFA) for 15 min and treated with 0.1% Triton X-100 for 5 min. After being blocked in PBS containing 5% FBS and 0.3% Triton X-100 for 1 h, the cells were incubated at 4°C overnight with the anti-IE antibody (a gift from E-S Huang) (14). The cells were rinsed and washed three times with PBS and incubated with the Alexa Fluor 594-conjugated anti-mouse IgG (Thermo Fisher Scientific) for 1.5 h. Finally, the nuclei were stained with 4′ 6-diamidino-2-phenylindole (DAPI). To observe *M. massiliense* infection, *M. massiliense* cells were stained with a 5 μM of Vybrant CFDA-SE (CFSE) cell tracker kit (Thermo Fisher Scientific) for 30 min at 37°C, washed two times and suspended in complement media before infection. Fluorescence was observed under a Leica TCS SP8 confocal microscope (Leica Microsystems). For quantification of the infection burden, the number of bacteria per cell was counted in three individual images per sample.

### Flow Cytometry

The HCMV TB40E-infected THP-1 cells and PBMCs were analyzed using an LSRFortessa^TM^ X-20 (BD Biosciences). PBMCs were stained with following antibodies; monoclonal antibodies to human CD45 (HI30), CD3 (HIT3a), CD19 (HIB19), CD11b (ICRF44), and CD14 (MfP9) all obtained from BD Biosciences. Data were analyzed using the FlowJo Software (version 7.6.2).

### Quantitation of HCMV Copy Number

The copy number of HCMV was determined by real-time polymerase chain reaction (PCR). Sample DNA was prepared with Media kit (Qiagen) from HCMV-infected THP-1 cell lysates according to the manufacturer’s recommendation. Real-time PCR reactions were performed using a Taqman MasterMix (Applied Biosystems) with 10 pmol HCMV US17 primer pairs, (5′-GCG TGC TTT TTA GCC TCT GCA-3′) and (5′-AAA AGT TTG TGC CCC AAC GGT A-3′), 10 pmol probe, FAM-5′-TGA TCG GCG TTA TCG CGT TCT TGA TC-3′-TAMRA, and sample DNA in a 20 μL reaction with an ABI QuantStudio 5 sequence detection system (Applied Biosystems). Primers and probes were synthesized commercially at Bioneer (Daejeon, South Korea). Reactions were performed under standard universal reaction conditions; hot start cycle of 10 min at 95°C, and 40 cycles of denaturation for 10 s at 95°C and annealing and extension for 60 s at 60°C. Standard curve for the quantitation of HCMV was obtained with the serially diluted samples of the known amount of HCMV.

### Gene Expression Analysis and RNA Sequencing

Total RNA was solubilized in TRIzol reagent (Invitrogen) and extracted according to the manufacturer’s instructions. Messenger RNA was reverse transcribed into cDNA with reverse transcription kits (Enzynomics) and quantitative real-time PCR for *UL111A* and *IL10* were performed using TaqMan PCR PreMix or SYBR Green PCR PreMix (Enzynomics) on an ABI PRISM 7900 (Applied Biosystems). The primer sequences used were as follows: *UL111A* (hcmvIL-10) forward: 5′-TGT TGA GGC GGT ATC TGG AGA-3′; *UL111A* reverse: 5′-CCG TCT TGA GTC CGG GAT AG-3′; *UL111A* probe: 5′-CCG GTT TCC CGC AGG CGA CC-3′; *IL10* forward: 5′-GCC TAA CAT GCT TCG AGA TC-3′; *IL10* reverse: 5′-TGA TGT CTG GGT CTT GGT TC-3′. For RNA sequencing analysis, total RNA was isolated, and the library preparation was performed using the NGS service provided by Ebiogen Inc. (Seoul, South Korea). In brief, each 500 ng total RNA were prepared and an oligo-dT primer containing an Illumina-compatible sequence at its 5′ end was hybridized to the RNA and reverse transcription was performed. After degradation of the RNA template, second strand synthesis was initiated by a random primer containing an Illumina-compatible linker sequence at its 5′ end. The double-stranded library was purified by using magnetic beads to remove all reaction components. The library was amplified to add the complete adapter sequences required for cluster generation. The finished library is purified from PCR components. High-throughput sequencing was performed as single-end 75 sequencing using NextSeq 500 (Illumina). QuantSeq 3′ mRNA-Seq reads were aligned using Bowtie2 (15). Bowtie2 indices were either generated from genome assembly sequence or the representative transcript sequences for aligning to the genome and transcriptome. The alignment file was used for assembling transcripts, estimating their abundances and detecting differential expression of genes. Differentially expressed gene were determined based on counts from unique and multiple alignments using coverage in Bedtools (16). The RC (Read Count) data were processed based on quantile normalization method using EdgeR within R (17) using Bioconductor (18). Gene classification was based on searches done by DAVID^1^, and genes that changed at least fourfold (*p* < 0.01) were analyzed for enrichment of gene ontology (GO) biological processes. For the hierarchical clustering and GO analysis, we used the MeV software and applied Euclidean distance and average linkage clustering for obtaining hierarchical clustering. Enriched terms that passed FDR < 20% were reported. For the Gene Set Enrichment Analysis (GSEA) for gene expression data, Molecular Signatures Database (V7.0) was used based on computing overlaps with GO gene sets (C5), obtained from the Broad Institute. All sequencing data can be found at the Gene Expression Omnibus (GEO) database (GEO accession number: GSE141236).

### ELISA

Culture supernatants were collected, centrifuged at 587 *g* for 5 min to remove particulates, and stored at −80°C until ELISA was performed. Human IL-10 was measured using the ELISA Duoset system (R&D Systems) according to the manufacturer’s instruction.

### Statistical Analysis

The Student’s *t*-test, one-way ANOVA or two-way ANOVA test were performed to determine statistically significant differences between groups using the GraphPad Prism 6 (GraphPad Software). Bonferroni’s or Tukey’s multiple comparisons test between all possible combinations were performed as post-tests for one-way ANOVA or two-way ANOVA, respectively. A value of *p* < 0.05 was deemed to be statistically significant.

## Results and Discussion

**FIGURE 1:**
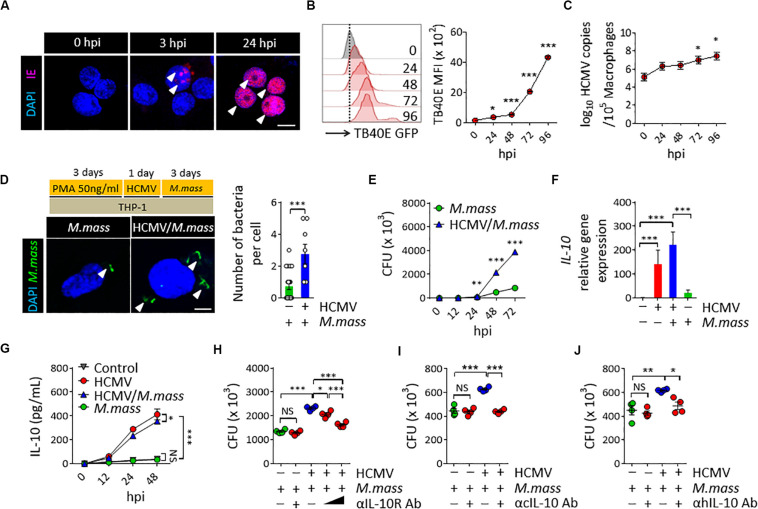
HCMV promotes *M. massiliense* proliferation in macrophages via increasing host IL-10 production. **(A)** Immunofluorescence staining for the HCMV IE expression. THP-1 macrophages were infected with HCMV at a MOI of 10 for the indicated time periods. hpi, hours post infection. Scale bar = 10 μm. **(B)** Offset histogram for the GFP expression intensities of HCMV-TB40E-GFP infected cells obtained using flow cytometry. Quantitative graph is shown on the right side. MFI, mean fluorescence intensity. **(C)** HCMV copy numbers determined by real-time PCR. **(D)** CFSE-labeled *M. massiliense* (MOI = 2) was used to infect the control and HCMV-infected THP-1 cells; the cells were examined at 24 h post *M. massiliense* infection under a fluorescence microscope. Representative images are shown. Number of bacteria per cell is quantitated in the left graph. Scale bar = 5 μm. **(E)** Quantification of the colony-forming units (CFUs) of *M. massiliense* at the indicated time points. **(F)** *IL10* gene expression was determined using real-time PCR. **(G)** IL-10 in the culture supernatants was measured using ELISA. **(H–J)** CFUs of *M. massiliense* at 48 hpi. HCMV was infected at 24 h prior *M. massiliense* challenge. Neutralization antibodies for the anti-IL-10 receptor antibody (αIL-10R Ab, 1 and 10 μg/mL), cmvIL-10 (αcIL-10 Ab, 10 μg/mL), human IL-10 antibodies (αhIL-10 Ab, 10 μg/mL) or corresponding isotype controls were added at 3 h post HCMV infection. All data are representative of three independent experiments. In panels **B,C,F,H,I,J**, one-way ANOVA was performed. In panel **D**, Student *t*-test was performed. In panels **E,G**, two-way ANOVA was used. *p < 0.05, **p < 0.01, ***p < 0.001. *N* = 3–7 biological replicates.

To establish HCMV infection in macrophages, THP-1 cells differentiated using PMA for 3 days (THP-1 macrophages) were infected with the HCMV TB40E strain at a MOI of 10, which expresses enhanced green fluorescence protein (GFP) at the tegument. As a result, we detected spot punctured IE expressions at the nucleus of THP-1 cells from 3 h post infection (hpi), and their further dissemination into the entire nucleus within 24 hpi (Figure 1A). HCMV efficiently replicated both in THP-1 macrophages (Figure 1B) and monocytes of human peripheral blood mononuclear cells (PBMCs) (Supplementary Figures S1A,B) from 24 to 96 hpi, as determined by analyzing the GFP intensity using flow cytometry and the viral copy numbers within THP-1 macrophages using real-time PCR (Figure 1C). Next, we examined the effect of HCMV infection in macrophages on the proliferation of *M. massiliense* infection. Uptake efficiency of *M. massiliense* infection (MOI = 2) for the initial 3 h of incubation were similar between the control and the HCMV Towne strain-infected macrophages (data not shown). However, we noticed that HCMV-infected macrophages had more intracellular bacteria from 24 hpi, and showed obviously increased bacterial burden at 72 hpi as determined by fluorescent particle numbers in the cells under a fluorescence microscope and by counting the CFUs of *M. massiliense* in the cell lysates (Figures 1D,E).

To investigate whether HCMV uses IL-10 signaling to promote *M. massiliense* proliferation in macrophages, we first examined the *UL111A* gene transcripts, which encode the cmvIL-10 in the HCMV-infected macrophages, using real-time PCR. As expected, HCMV infection induced *UL111A* transcription at 12 hpi (Supplementary Figure S2A). We next analyzed the expression of human IL-10 and found that HCMV infection significantly elicited host IL-10 synthesis both in THP-1 macrophages (Figures 1F,G) and PBMCs (Supplementary Figure S2B). Treatment of recombinant cmvIL-10 (Supplementary Figures S2C–E) or culture supernatant of HCMV-infected macrophages (Supplementary Figures S2F,G) directly induced human IL-10 synthesis, and the presence of neutralizing antibodies for the cmvIL-10 during HCMV infection significantly reduced the host IL-10 synthesis (Supplementary Figures S2H,I), suggesting that cmvIL-10 secreted from HCMV-infected macrophages elicited host IL-10 production. In particular, *M. massiliense* co-infection could not change markedly IL-10 production by HCMV-infected macrophages (Figure 1F), and we observed a clear distinction between IL-10 synthesized by HCMV-infected macrophages versus that by non-infected macrophages in the presence and absence of *M. massiliense* co-infection (Figure 1G), implicating the dominant immunomodulatory effects of HCMV infection, excluding the effects of challenges with other bacteria.

**FIGURE 2:**
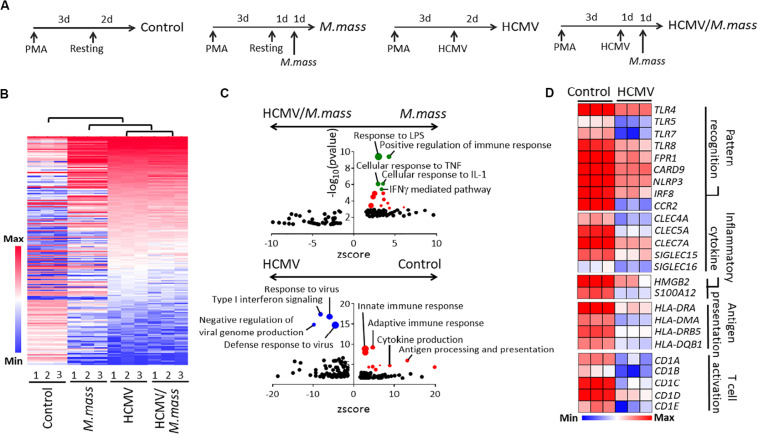
HCMV infection blocks pro-inflammatory and anti-bacterial immune responses in macrophages. **(A)** Schematic figure for each experimental groups for RNA sequencing; Control, *M. massiliense*-infected (MOI = 2, 24 h), HCMV-infected (MOI = 10, 48 h), and HCMV/*M. massiliense* co-infected. Triplicates per group from a single experiment were used. **(B)** Hierarchical clustering analysis showed a high similarity between HCMV- and HCMV/*M. massiliense*-infected cells compared to *M. massiliense*-infected cells. **(C)** Biological processes enriched in the gene ontology analysis of *M. massiliense*- versus HCMV/*M. massiliense*- (Upper) and control versus HCMV-infected macrophages (Bottom) are shown as GO plots. **(D)** Heatmaps showing differentially expressed genes associated with pattern recognition, inflammatory cytokine, antigen presentation, and T cell activation in the control and HCMV-infected cells.

To evaluate the impact of IL-10 on the proliferation of *M. massiliense*, we neutralized the effect of endogenous human IL-10 by adding anti-IL-10 receptor neutralizing antibody (αIL-10R Ab) to the culture supernatant of *M. massiliense*-infected macrophages. As a result, bacterial burden decreased in an αIL-10R Ab dose-dependent manner in the HCMV-infected THP-1 macrophages (Figure 1H) as well as in PBMCs (Supplementary Figure S2J). Neutralization of IL-10R did not affect *M. massiliense* proliferation without a precedent HCMV infection. Adding neutralization antibodies for the cmvIL-10 (αcIL-10 Ab) or human IL-10 (αhIL-10 Ab) also decreased bacterial burden in the HCMV-infected THP-1 macrophages (Figures 2I,J) and PBMCs (Supplementary Figures S2K,L), further supporting the notion that HCMV inhibits host anti-bacterial activities by modulating IL-10 production via cmvIL-10. Collectively, these results demonstrate that HCMV modulates macrophage immune response to aggravate a subsequent coinfection with *M. massiliense* via inducing host IL-10 production.

Next, we profiled the transcriptome of HCMV-, HCMV/*M. massiliense*- and *M. massiliense-*infected macrophages (Figure 2A). First, HCMV-infected macrophages showed the 880 differentially expressed genes (DEGs, 500; upregulated, 380; downregulated) compared to control macrophages (Supplementary Table S1). Based on these DEGs, we obtained hierarchical clustering between four groups and found that *M. massiliense* co-infection did not globally change the transcriptome of HCMV-infected macrophages (Figure 2B). The presence of replicating HCMV in macrophages brought low immune responsiveness upon secondary *M. massiliense* challenge, which was clearly shown in the GO analysis. *M. massiliense* single-infected macrophages showed enriched GO pathways including response to lipopolysaccharide (LPS) and cellular response to TNF/IL-1 compared to GO pathways enriched in the co-infected group (Figure 2C and Supplementary Table S2) obtained from DAVID software. Gene Set Enrichment Analysis (GSEA) showed similar results that *M. massiliense* single-infected macrophages had enriched GO pathways including positive regulation of immune response compared to co-infected group (Supplementary Table S3). To support this result, GO pathways of the innate immune response, adaptive immune response, and antigen processing were enriched in the downregulated DEGs of HCMV-infected macrophages, compared to those in the control macrophages. Signature innate immune response genes, such as *TLRs, NLRP3, CLECs, HLAs*, and *CDs*, were strongly downregulated upon HCMV infection (Figure 2D), demonstrating that the global programming of macrophages into anti-inflammatory phenotype had occurred through HCMV infection.

Initial response to pathogens of the innate immune cells determines the direction of systemic immune responses and consequent disease outcome (19). HCMV establishes a lifelong persistence in cells of myeloid lineages, affecting various immune responses of hosts when reactivated (3). Estimated seroprevalence of HCMV is now 45–100% in the general population depending on the geographic location and the socio-economic status (20), resulting in an important need to study the possible effect of HCMV infection on emerging problematic infections. Cobelens et al. (11) recently reported that HCMV infection plays an epidemiologically relevant role in the etiology of tuberculosis by furthering the progression from latent tuberculosis infection to disease. One possible mechanism for this phenomenon is that an elevated type I IFN associated with susceptibility to bacterial infections including *Mycobacterium tuberculosis* (21–23), also detected in this study, is upregulated by HCMV infection. Besides the general IFN response to virus infection, we have shown here that HCMV boosted IL-10 production by host macrophages, and this phenomenon potentially altered the anti-bacterial defense mechanism. IL-10 can block phagosome maturation (24) and inhibit the production of pro-inflammatory cytokines (25, 26), resulting in macrophage deactivation, and preventing the release of reactive nitrogen species by macrophages (27). Prominent IL-10 synthesis during HCMV infection, therefore, induces the formation of the anti-inflammatory phenotype of the macrophages, finally reducing their ability to restrict *M. massiliense* proliferation. Although there are several conflicting reports insisting that the M1 polarization of macrophages is caused by HCMV (28–30), this study explains the increased burden of NTM in HCMV-infected macrophages via host IL-10 modulation.

## Data Availability Statement

The datasets generated for this study can be found in the GSE141236 / https://www.ncbi.nlm.nih.gov/geo/query/acc.cgi?acc=GSE141236.

## Author Contributions

SHS, HQ, YRN, and JK conceived and designed the research study, performed the experiments, analyzed the data, and wrote the manuscript. YRN, JHK, By-JK, and Bu-JK discussed and commented on the manuscript. ESH, SHS, and JJH designed the research study and supervised the manuscript. All authors reviewed the manuscript before submission.

## Conflict of Interest

The authors declare that the research was conducted in the absence of any commercial or financial relationships that could be construed as a potential conflict of interest.

## Footnotes

1 http://david.abcc.ncifcrf.gov/

## Supplementary Material

FIGURE S1 HCMV replicates in human monocytes. **(A)** Contour plots for the gating strategy. PBMCs isolated from healthy volunteers were infected with HCMV TB40E strain at a MOI of 10. CD45^+^CD14^+^CD11b^+^ monocytes, CD45^+^CD3^–^CD19^+^ B cells and CD45^+^CD3^+^CD19^–^ T cells were further analyzed for the expression of GFP at 72 h post infection. Representative from three different donors. **(B)** Quantitative graph indicating the GFP expression intensities in each population assessed by flow cytometry. *N* = 2. MFI, mean fluorescence intensity. One-way ANOVA was performed. ^∗^*p* < 0.05, ^∗∗∗^*p* < 0.001.

FIGURE S2 HCMV promotes *M. massiliense* proliferation in THP-1 macrophages and PBMCs via increasing host IL-10 production. **(A)** *UL111A* gene expression was examined at 12 h post infection (hpi) with *M. massiliense* (MOI = 2) using real-time PCR in THP-1 macrophages. HCMV was infected (MOI = 10) at 24 h prior to *M. massiliense* infection. *N* = 6. **(B)** IL-10 in the culture supernatant of PBMCs at 48 hpi was measured using ELISA. *N* = 2. **(C)** *IL10* gene expression was determined using real-time PCR in THP-1 macrophages treated with 200 ng/mL of cmvIL-10 for 12 h. *N* = 6. **(D)** IL-10 in the culture supernatant of THP-1 macrophages was measured using ELISA. *N* = 2. **(E)** IL-10 in the culture supernatant of PBMCs treated with 200 ng/mL of cmvIL-10 for 48 h was measured using ELISA. *N* = 2. **(F)** *IL10* gene expression at 12 hpi was determined using real-time PCR. Culture media of differentiated THP-1 macrophages was changed with HCMV culture supernatant at 24 h prior to *M. massiliense* infection. *N* = 3. **(G)** IL-10 in the culture supernatant of THP-1 macrophages at 48 hpi was determined by ELISA. Culture media of differentiated THP-1 macrophages was changed with HCMV culture supernatant at 24 h prior to *M. massiliense* infection. *N* = 3. **(H,I)** IL-10 in the culture supernatant of THP-1 macrophages **(H)** and PBMCs **(I)** was determined by ELISA. Neutralization antibodies for the cmvIL-10 (αcIL-10 Ab, 10 μg/mL) or corresponding isotype controls were added at 3 h post HCMV infection. *N* = 2. **(J–L)** CFUs of *M. massiliense* at 48 hpi in PBMCs. HCMV was infected at 24 h prior *M. massiliense* challenge. A 10 μg/mL of neutralization antibodies for the anti-IL-10 receptor antibody (αIL-10R Ab), cmvIL-10 (αcIL-10 Ab), human IL-10 antibodies (αhIL-10 Ab) or corresponding isotype controls were added at 3 h post HCMV infection. *N* = 4. All data are representatives of two independent experiments. For the PBMCs, representative data obtained from two independent donors are shown. In all panels, except panel **(E)**, one-way ANOVA was performed. In panel **(E)**, Student *t*-test was performed. ^∗^*p* < 0.05, ^∗∗^*p* < 0.01, ^∗∗∗^*p* < 0.001. *N* = 3–7 biological replicates.
