# Supplementary figures and images for: Human Cytomegalovirus-Induced Interleukin-10 Production Promotes the Proliferation of Mycobacterium massiliense in Macrophages
Source: Front Immunol. 2020 Sep 10;11:518605. doi: 10.3389/fimmu.2020.518605 (PMC7511582; doi:10.3389/fimmu.2020.518605)

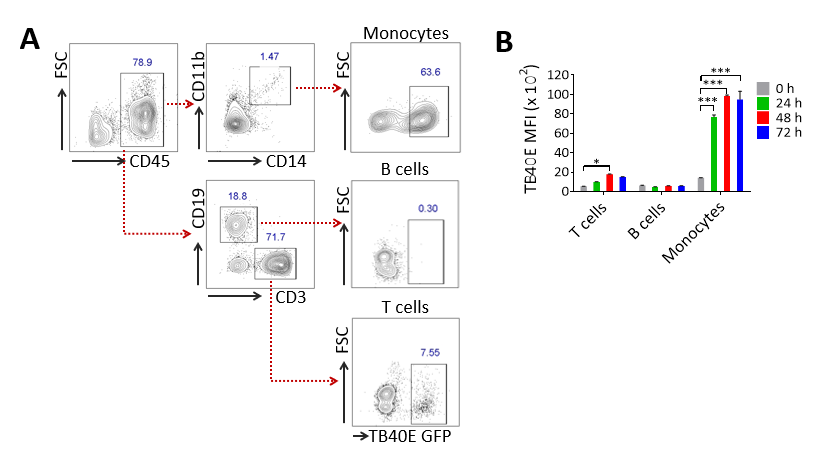

Supplement: FIGURE S1 — HCMV replicates in human monocytes. (A) Contour plots for the gating strategy. PBMCs isolated from healthy volunteers were infected with HCMV TB40E strain at a MOI of 10. CD45+CD14+CD11b+ monocytes, CD45+CD3–CD19+ B cells and CD45+CD3+CD19– T cells were further analyzed for the expression of GFP at 72 h post infection. Representative from three different donors. (B) Quantitative graph indicating the GFP expression intensities in each population assessed by flow cytometry. N = 2. MFI, mean fluorescence intensity. One-way ANOVA was performed. ∗p < 0.05, ∗∗∗p < 0.001. [file Image_1.tif]

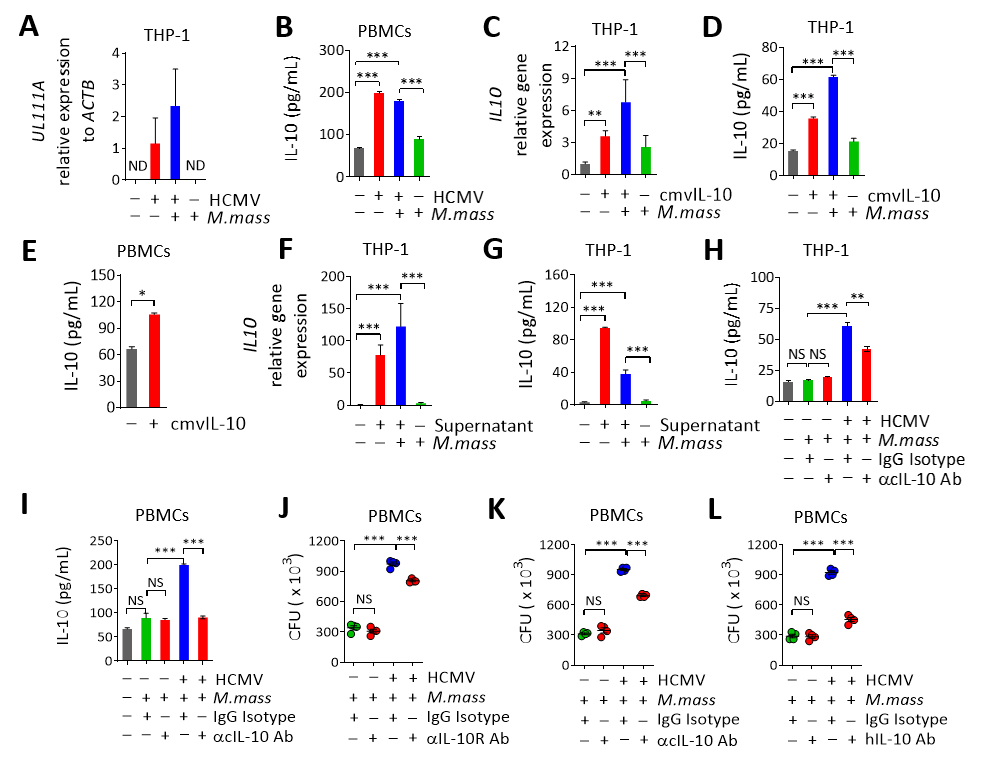

Supplement: FIGURE S2 — HCMV promotes M. massiliense proliferation in THP-1 macrophages and PBMCs via increasing host IL-10 production. (A) UL111A gene expression was examined at 12 h post infection (hpi) with M. massiliense (MOI = 2) using real-time PCR in THP-1 macrophages. HCMV was infected (MOI = 10) at 24 h prior to M. massiliense infection. N = 6. (B) IL-10 in the culture supernatant of PBMCs at 48 hpi was measured using ELISA. N = 2. (C) IL10 gene expression was determined using real-time PCR in THP-1 macrophages treated with 200 ng/mL of cmvIL-10 for 12 h. N = 6. (D) IL-10 in the culture supernatant of THP-1 macrophages was measured using ELISA. N = 2. (E) IL-10 in the culture supernatant of PBMCs treated with 200 ng/mL of cmvIL-10 for 48 h was measured using ELISA. N = 2. (F) IL10 gene expression at 12 hpi was determined using real-time PCR. Culture media of differentiated THP-1 macrophages was changed with HCMV culture supernatant at 24 h prior to M. massiliense infection. N = 3. (G) IL-10 in the culture supernatant of THP-1 macrophages at 48 hpi was determined by ELISA. Culture media of differentiated THP-1 macrophages was changed with HCMV culture supernatant at 24 h prior to M. massiliense infection. N = 3. (H,I) IL-10 in the culture supernatant of THP-1 macrophages (H) and PBMCs (I) was determined by ELISA. Neutralization antibodies for the cmvIL-10 (αcIL-10 Ab, 10 μg/mL) or corresponding isotype controls were added at 3 h post HCMV infection. N = 2. (J–L) CFUs of M. massiliense at 48 hpi in PBMCs. HCMV was infected at 24 h prior M. massiliense challenge. A 10 μg/mL of neutralization antibodies for the anti-IL-10 receptor antibody (αIL-10R Ab), cmvIL-10 (αcIL-10 Ab), human IL-10 antibodies (αhIL-10 Ab) or corresponding isotype controls were added at 3 h post HCMV infection. N = 4. All data are representatives of two independent experiments. For the PBMCs, representative data obtained from two independent donors are shown. In all panels, except panel (E), one-way ANOVA was per [file Image_2.tif]
